# Effects of Gender and Age in Mandibular Leeway Space for Taiwanese Children

**DOI:** 10.3390/children8110999

**Published:** 2021-11-03

**Authors:** Kuo-Ting Sun, Yun-Zhen Wu, Jui-Ting Hsu, Min-Chia Tsai, Heng-Li Huang

**Affiliations:** 1 School of Dentistry, College of Dentistry, China Medical University, Taichung 404, Taiwan; sunny19670823@gmail.com (K.-T.S.); jthsu@mail.cmu.edu.tw (J.-T.H.); 2 Department of Pediatric Dentistry, China Medical University Hospital, Taichung 404, Taiwan; b50703@gmail.com; 3 Department of Biomedical Imaging and Radiological Science, China Medical University, Taichung 404, Taiwan; jenny890701@gmail.com; 4 Department of Bioinformatics and Medical Engineering, Asia University, Taichung 412, Taiwan

**Keywords:** leeway space, mesiodistal crown width, mixed dentition, permanent dentition

## Abstract

Purpose: Leeway space is clinically crucial in pediatric dentistry because it is utilized to resolve tooth crowding and allow the first molars to drift mesially to establish a Class I molar relationship in the later stages of mixed dentition. This study investigated leeway space in the mixed dentition of Taiwanese children of different sexes and ages. Materials and Methods: The digital panoramic dental films of 182 lower arches of 119 boys and 63 girls aged 5–10 years were analyzed in this retrospective study. The mesiodistal crown widths of the primary canines and first and second molars and the permanent canines and first and second premolars were measured using medical imaging software. Differences in leeway space were statistically analyzed. Results: The average leeway space was 1.29 ± 1.48 mm on each side of the lower arch. The leeway space of children aged 5–6 years was significantly greater than that of children aged 7–8 years. No gender difference in crown width was discovered, except with regard to the primary first molar. Although no gender difference in leeway space was observed, permanent teeth affected leeway space more for girls than for boys. Conclusion: In Taiwanese children, although leeway space does not differ by sex, age affects leeway space. However, permanent tooth size has an influence on the leeway space of girls.

## 1. Introduction

Leeway space, the most crucial space for the alignment of permanent teeth, is the difference between the sum of the mesiodistal crown widths of the primary canines and molars and that of their successors, the permanent canines and premolars [1]. Primary molars have greater mesiodistal crown widths than the permanent premolars that replace them, especially the primary second molar [2]. Leeway space has been preserved and utilized to resolve crowding [3], particularly by allowing the first molars to drift mesially to establish a Class I molar relationship in the late mixed dentition [4,5].

Leeway space has been reported to be affected by many factors, including ethnicity, [6] the crown size of primary [7] and permanent teeth [8], sex [9], and environmental factors [8]. The average leeway space was reported to be 0.9 and 1.8 mm per quadrant for the maxilla and mandible, respectively, in the United States [10]. Leeway space was reported to be 2.03 mm in the lower arch in Brazil [2]. Permanent teeth were demonstrated to have a secular trend of growing, leading to a significant reduction in leeway space [8]. Girls have more leeway space than boys have [9]. Environmental factors that reduce tooth crown dimensions may affect leeway space [11].

Leeway space is more crucial in the mandibular arch than in the maxillary arch; the therapeutic choices for the mandibular arch are limited because of the low potential for arch expansion and the difficulty of molar distalization [3,8] By contrast, spatial problems in the maxillary arch can be resolved through arch expansion, buccal tipping of the anterior segments, and molar distalization. As a result, the leeway space in the maxilla was negative in several cases [9]. Therefore, several studies have investigated leeway space in the mandibular arch [3,8] and utilizing leeway space in the mandible is one of the few means of resolving anterior arch crowding [4].

The investigation of leeway space is valuable for predicting the integrity of the dental arch and the first molar relationship, but no studies have focused on leeway space in Asia. This study investigated (1) the value of leeway space in the mandibular arch; (2) whether the crown widths of the primary canines and molars and permanent canines and premolars influence leeway space; and (3) whether leeway space differs by gender.

## 2. Materials and Methods

### 2.1. Patient Selection

The digital panoramic dental films of 182 lower arches of 119 boys and 63 girls were analyzed in this retrospective study. The children were aged 5 to 10 years. The inclusion criterion was complete and normal primary dentition, and the exclusion criteria were crowding, congenital missing teeth, a premature loss of primary teeth, dental caries, and abnormal morphology. Clear digital panoramic images were used to measure the crown widths of the primary canines and first and second molars and the permanent canines and premolars. The images were also used to confirm that the eruption and development of permanent teeth were normal and well aligned; that no crowding, congenital missing teeth, or supernumerary teeth were present; and that morphology was normal. The exclusion criteria were the abnormal eruption of primary or permanent teeth, proximal caries, crowding, congenital missing teeth, and other abnormalities such as supernumerary teeth or unclear digital images. This study was approved by the Institutional Review Board of China Medical University Hospital (CMUH109-REC3-059). The ethic approval date was 6 May 2020.

### 2.2. Digital Panoramic Scanning and Measurement of Leeway Space

Digital panoramic radiography was performed by a pediatric dentist in our pediatric dental clinic. The AutoIIIN panoramic radiograph (Asahi Roentgen Ind. Co., Ltd., Kyoto, Japan) was used. The panoramic films were developed using a FireCR Dental Reader (Digiray Corp., Gyeonggi-do, Republic of Korea). According to the hospital’s standard regulation for taking panoramic films, the head position of patients must be straight and not tilted and the Frankfort plane kept parallel with the floor. The upper and lower incisors need to be placed correctly on the bite block, and the lips kept together for the duration of the exposure. The X-ray energy setting was 70 kV and 8 mA provides the optimum exposure condition. The exposure time was 12 s.

**Figure 1:**
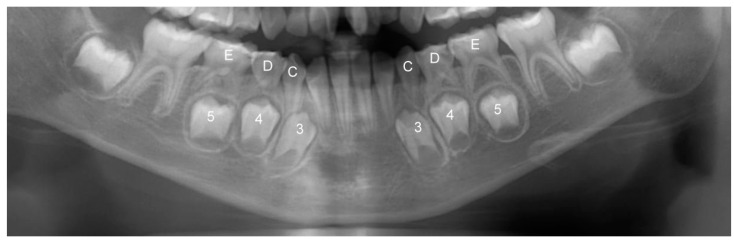
Panoramic image of deciduous teeth and permanent teeth in the lower jaw of one patient. According to clinical naming convention, 3, 4, and 5 indicates primary canines and first and second molars as well as C, D, and E represents permanent canines and two premolars. The difference between the mesiodistal crown widths of 345 and CDE is the value of leeway space.

The size and width of the teeth in the mandible were measured using EZ Dental System software (Fu Ji Instrument Co., Ltd., Taichung, Taiwan). The measuring system was regulated and calibrated regularly, and the value measured was the true width of the tooth crown. To maintain the accuracy of statistics, each tooth was measured individually at its maximal width, and the teeth in the image that had overlapping imaging problems were not included. The mesiodistal crown width of the primary canines and first and second molars and the permanent canines and two premolars were measured and recorded (Figure 1). The differences in the mesiodistal crown width of the primary canines and the first and second molars and the permanent canines and two premolars were calculated as leeway space. The measurements were approved by K.T. Sun, who is a senior professional pediatric dentist.

### 2.3. Statistical Analysis

The Shapiro–Wilk test was performed to confirm that the data were normally distributed. The Student’s *T* test was performed to compare leeway space by sex. The data were divided into groups of participants aged 5–6, 7–8, or 9–10 years. Analysis of variance was used to compare the data among the age groups. Scheffe’s post hoc test was performed for pairwise comparisons. Bivariate analysis (Pearson correlation) was also performed to evaluate the correlation of primary and permanent tooth widths with leeway space. All statistical analyses were performed using SPSS (SPSS Inc., Chicago, IL, USA), with the significance level set at *p* = 0.05.

## 3. Results

The leeway space in the lower arches is presented in Table 1. The average leeway space was 1.29 ± 1.48 mm on each side of the lower arch. The averages by sex were 1.17 ± 1.48 mm for boys and 1.53 ± 1.45 mm for girls, but the difference was not significant.

The mesiodistal crown widths of the primary canines and first and second molars and the permanent canines and first and second premolars are presented in Table 2. No significant difference between sexes was observed, except in the lower primary first molar. The crown of the primary first molar was wider in girls than in boys. 

No significant differences in leeway space were observed between children aged 5–6 and 9–10 years or between those aged 7–8 and 9–10 years (Table 3). However, the leeway space of children aged 5–6 years was significantly greater than that of children aged 7–8 years (Table 3).

Leeway space had a significant and positive correlation with primary tooth width and a negative correlation with permanent tooth width for all participants and for boys (Table 4). For girls, leeway space was significantly correlated with permanent tooth width but not with primary tooth width (Table 4).

## 4. Discussion

In the late mixed dentition, a loss of leeway space can cause dental crowding and malocclusion, and orthodontic treatment may be required. Therefore, preserving leeway space is crucial during the transition from the primary to permanent dentition. To our knowledge, this might be the first study related to leeway space in Asia. We determined the average leeway space to be 1.29 ± 1.48 mm on either side of the lower arch. No gender differences in leeway space were observed. Children aged 5–6 years had significantly greater leeway space than did those aged 7–8 years. Leeway space was significantly related to the crown width of primary and permanent teeth, except for the primary first molars of girls.

A lingual holding arch was used to preserve leeway space in the mandibular arch [12]. In the maxillary arch, the transpalatal arch and Nance appliance were used to preserve leeway space [13]. An unfavorable eruption sequence reduces the available space, and a lingual arch is recommended to preserve leeway space [14]. Because of the many functions of leeway space, if leeway space is prematurely lost, it must be regained. Crowding in the maxillary arch can be resolved through several methods, including arch expansion, buccal tipping of the anterior segments and molar distalization [9,15]. However, for the mandibular arch, the therapeutic choices are limited [3] and molar distalization is difficult. In one study, maxillary teeth were approximately equal to their primary counterparts [16]. Therefore, leeway space is more critical for the lower jaw than for the maxilla. This is why the presented study demonstrates the importance of leeway space, especially in the mandibular arch, during the transition from the primary to permanent dentition.

Earlier studies have reported that the measurement of average leeway space in mandible was between 1.8 mm and 3.6 mm [1,2,8,9,10]. Compared with the previous studies above, this study observed less leeway space in the mandible. Several factors may have influenced this result. First, most previous studies have been conducted outside of Asia, in regions such as the United States [8,10], South America [2,9] and others [1]. It looks as though ethnicity affects leeway space. Second, leeway space is determined by the crown width of the primary canines and first and second molars and the permanent canines and premolars. According to this study, leeway space was closely related to the crown size of permanent teeth. The sum of the crown widths of the permanent canines and premolars in this study was greater than those reported in previous studies in the United States, Mexico and the United Kingdom [9,17,18,19]. Larger crown diameters of permanent teeth might result in less leeway space. Furthermore, positive secular trends in the crown widths of permanent teeth have been observed [8,20]. This phenomenon could reduce leeway space.

In some studies, leeway space has been greater in girls than boys [9,21]. However, some researchers have found no gender differences in leeway space [8]. In this study, no significant differences between sexes were observed. The reason for the larger leeway space of girls in previous studies may have been the narrower crowns of the girls’ permanent teeth, especially the canines, than those of the boys [9,18]. Narrower permanent crowns may be associated with greater leeway space. However, in the present study, when the crown widths of the boys’ and girls’ permanent and primary teeth were compared, there were no significant differences between sexes. Therefore, this might be the reason that the reason leeway space did not differ by gender in Taiwanese children.

In the present study, the leeway space of children aged 5–6 years was significantly greater than that of children aged 7–8 years, and the total crown size of their permanent teeth was significantly smaller. This may explain why children aged 5–6 years had significantly more space than those aged 7–8 years. The timing of tooth eruption from the jawbone is correlated with crown size, and smaller teeth germinate earlier than larger teeth. The smaller total crown size of the permanent teeth of children aged 5–6 years resulted in significantly more leeway space than in the group aged 7–8 years.

With larger primary crowns or smaller permanent crowns, leeway space is greater. In this study, leeway space had a positive correlation with the crown sizes of primary teeth and a negative correlation with the crown sizes of permanent teeth. In boys and all participants (not divided by sex), leeway space was correlated with primary and permanent tooth widths. However, in girls, leeway space had a higher correlation with permanent tooth width than primary tooth width. This indicates that, for girls, the size of permanent teeth affected leeway space more than that of primary teeth because the gender difference in permanent tooth size is greater than the primary tooth size.

There are some limitations in this study. First, the study results would have been more representative had more children been included. Second, the dental panoramic radiography was probably slightly distorted because of differences in head positioning. Cone-beam computed tomography can scan all teeth, including erupted and unerupted teeth, without magnification or distortion. Third, leeway space is influenced by many factors, including genetics, dental problems, and other factors. Further studies should investigate such factors to further understand leeway space.

## 5. Conclusions

Despite the limitations, the following conclusions were drawn:

1. The average leeway space was 1.29 ± 1.48 mm on each side of the lower arch.
2. The leeway space of children aged 5–6 years was significantly greater than that of children aged 7–8 years.
3. No gender differences in mandibular leeway space were found.
4. Permanent teeth had a greater impact on leeway space for girls than for boys.

## Figures and Tables

**Table 1:** Leeway space (mm) for all children and boy and girl groups.

	Mean	SD	Max.	Min.
Whole	1.29	1.48	6.45	−2.79
Boy	1.17	1.48	6.45	−2.79
Girl	1.53	1.45	4.33	−1.80

**Table 2:** Mesiodistal crown widths (mean ± standard deviation in mm) of primary and permanent teeth.

Tooth	Position	Whole	Boy	Girl	*p* Value
Primary teeth	canine	5.61 ± 0.66	5.57 ± 0.62	5.7 ± 0.74	0.07
	First molar	8.18 ± 1.01	8.17 ± 1.07	8.2 ± 0.88	0.05
	second molar	10.91 ± 1.25	10.88 ± 1.25	10.95 ± 1.24	0.75
Permanent teeth	canine	7.61 ± 1.02	7.71 ± 1.01	7.43 ± 1.02	0.65
	First premolar	7.92 ± 0.97	7.9 ± 0.99	7.95 ± 0.93	0.42
	second premolar	7.88 ± 0.93	7.84 ± 0.87	7.94 ± 1.04	0.28

Student’s *t* test was performed to compare the measurements between boys and girls. Bold font indicates statistical significance.

**Table 3:** Leeway space (mean ± standard deviation in mm) for various age groups *p* value < 0.05 †.

Age	Mean *	SD
5–6	1.92 ^a^	1.52
7–8	1.04 ^b^	1.37
9–10	1.2 ^ab^	1.47

† One way ANOVA. * *Scheffe post hoc* pairwise comparisons; mean in the same row with the same. letters (^a^, ^b^) do not significantly differ.

**Table 4:** Correlations between primary and permanent tooth width and leeway space for all children and boy and girl groups.

Group	Pearson Correlation Coefficient, r (*p*)	
	Primary Teeth	Permanent Teeth
Whole	0.28 (<0.001) *	−0.29 (<0.001) *
Boy	0.34 (<0.001) *	−0.22 (0.016) *
Girl	0.13 (0.309)	−0.42 (0.001) *

Note: * indicates statistical significance.

## Data Availability

The data that support the findings of this study are available from the corresponding author upon reasonable request.
